# Prediction and diagnosis of bladder cancer recurrence based on urinary content of *hTERT*, *SENP1*, *PPP1CA*, and *MCM5 *transcripts

**DOI:** 10.1186/1471-2407-10-646

**Published:** 2010-11-24

**Authors:** Anne Sofie Brems-Eskildsen, Karsten Zieger, Helle Toldbod, Cherie Holcomb, Russell Higuchi, Francisco Mansilla, Pia P Munksgaard, Michael Borre, Torben F Ørntoft, Lars Dyrskjøt

**Affiliations:** 1 Department of Molecular Medicine, Aarhus University Hospital, Skejby, Denmark; 2 Department of Urology, Aarhus University Hospital, Skejby, Denmark; 3 Department of Hematology, Aarhus University Hospital, Aarhus, Denmark; 4 Roche Molecular Systems, Pleasanton, California, USA

## Abstract

**Background:**

Identification of urinary biomarkers for detection of bladder cancer recurrence would be beneficial to minimize the frequency of cystoscopy. Our objective was to determine the usability of urine content of mRNA in the detection and prediction of bladder cancer recurrence.

**Methods:**

We analyzed 123 prospectively cross-sectional collected urine samples from 117 patients with bladder cancer (12 incident cancers and 111 control visits). We used biopsies from cystoscopies as diagnostic criteria for recurrence, and followed the patients for a median time of 28.5 months (range 0-44 months). We measured the levels of *hTERT, SENP1, PPP1CA*, and *MCM5 *mRNA in urine by q-RT- PCR.

**Results:**

We found significant differences in urinary content of *hTERT *(p < 0.001), *SENP1 *(p < 0.001), *MCM5 *(p < 0.001), and *PPP1CA *(p < 0.001) transcripts, when comparing urine samples from patients with and without tumor present in the bladder. We obtained sensitivity and specificity values for *hTERT*: 63/73, *SENP1*: 56/78, *MCM5*: 63/66, and *PPP1CA*: 69/63, respectively. Including follow-up data resulted in sensitivity and specificity values for *hTERT*: 62/84, *SENP1*:53/84, *MCM5*: 61/73, and *PPP1CA*: 65/66. Interestingly, at non-tumor visits the urinary content of especially *hTERT *(p = 0.0001) and *MCM5 *(p = 0.02) were significantly associated with subsequent tumour recurrence. Combining the markers with cytology improved the detection. The best combination was *hTERT *and cytology with a sensitivity of 71% and a specificity of 86% after follow-up. Further prospective validation or registration studies needs to be carried out before clinical use.

**Conclusions:**

We could use the urinary content of *hTERT*, *SENP1*, *PPP1CA*, and *MCM5 *to detect bladder cancer recurrence. All markers showed a higher sensitivity than cytology. The detection rate improved when including cytology results, but also the combination of *hTERT *and *MCM5 *increased the detection rate. Furthermore, *hTERT *and *MCM5 *levels predicted subsequent tumor recurrences.

## Background

Non-muscle invasive bladder cancer is characterized by frequent tumor recurrences. Today the standard follow-up consists of cystoscopy combined with cytological examination at an interval of 3 to 6 months depending on tumor malignancy and previous recurrence rate. Cystoscopic examinations are unpleasant, time consuming, expensive, and may have serious side effects such as infections and damage to urethra [1]. Cytology is characterized by a high specificity (0.83-0.997) but a low sensitivity (0.20-0.53) [2]. It has been calculated that bladder cancer invokes the highest cost per patient from diagnosis to death; in the US calculated to exceed 3.4 billion dollars annually [3]. Bladder tumor markers may reduce these costs significantly.

Current FDA approved assays available for bladder cancer detection are: BTA stat, BTA TRAK, NMP22, FDP, Urovysion, and ImmunoCyt. One of the best characterized and more promising biomarkers is telomerase (*hTERT*) [4-8]. *hTERT *is a ribonucleoprotein enzyme that maintains telomeric repeats at the end of the chromosomes in order to compensate for sequence loss during DNA replication [7]. Telomerase is inactive in mature cells, however, telomerase activity can be detected in 85-90% of all primary human cancers [9] and reactivation immortalize cells [10-12]. *hTERT *is described as superior to cytology in regard to sensitivity, but false positive results are seen in benign urothelial conditions [2,7].

Other potential biomarkers like *MCM5*, *PPP1CA*, and *SENP1 *are less thoroughly described. *MCM5 *drives the formation of pre-replicative complexes in the first key event during G1 phase and is essential for cell proliferation. *MCM5 *is detectable in urine and associated with adverse outcome [13,14]. *PPP1CA *is involved in *pRB *dephosphorylation [15,16] and ceramide accumulation induced by *RAS*, as a response to oncogenic stress increases *PPP1CA *activity, *pRB *phosphorylation and onset of *p53 *induced arrest, and thereby contributes to tumor suppression [15,16]. *SENP1 *is a SUMO-specific peptidase shown to desumorylate promyelocytic leukemia (PML) protein [17]. PML protein is a tumor suppressor protein requiring SUMOylation [18,19]. PML bodies of an altered morphology have been described in alternative lengthening of telomeres (ALT) [20]. *SENP1 *might function as a marker for tumors in which telomeres were maintained by ALT, as not all, especially invasive tumors, are *hTERT *positive [21].

Here we evaluated the diagnostic value of measuring the urinary content of *hTERT, MCM5, PPP1CA*, and *SENP1 *mRNAs for detection of tumor recurrence.

## Methods

### Patient material

Urine samples were collected prospectively at the Department of Urology at Aarhus University Hospital, Skejby. Informed consent was obtained from all patients involved, and the protocol was approved by the Scientific Ethics Committee in Aarhus County. We collected 50 mL urine at regular follow-up visits. All patients diagnosed with bladder cancer were included in the study. In total urine samples from: 54 patients with biopsy proven recurrent bladder cancer at sampling, 59 patients with a previous bladder cancer and no tumor at sampling, and 10 patients with a primary bladder cancer at sampling time. The tumor biopsies were graded according to Bergkvist (Table 1). Twenty-two of the patients received Bacille Calmette-Guérin (BCG), two patients received Mitomycin (MMC), and six patients received radiotherapy (Table 2). Eleven urine specimens were from patients with muscle invasive tumors.

**Table 1:** Distribution of tumor stage and grade among all included patients in the study

Grade and stage distribution	None	Grade 1	Grade 2	Grade 3	Total
No tumor	59				59
Ta		10	26	7	43
T1			1	5	6
T2-4			1	10	11
CIS				4	4

Total	59	10	28	26	123

**Table 2:** Patient and tumor characteristic for patients with recurrent and non-recurrent tumors

		Recurrent and primary	Non-recurrent	P-value (Mann-Whitney)
EORTC	1 year	0.33	0.29	0.12
Recurrence score* N = 99	5 years	0.55	0.51	0.13

EORTC	1 year	0.032	0.029	0.16
Progression score* N = 99	5 years	0.11	0.099	0.16

Age at urine sample (years)		69	71	0.18
Male/female ratio		57/20	35/11	
Follow-up time (months)		28	29	0.75
Leucocyte pos/neg ratio		5/68	5/40	
BCG or MMC treatment before urine sampling		13	11	0.82

Average pellet size**		1.39	1.41	0.95
Average RNA quality (RIN)		3.70	3.83	0.89
Average RNA conc. (pg/μL)		5995 (32-169186)	7990 (5-117671)	0.0029

* EORTC scores are only calculated for non-muscle invasive tumors. There are 51 urine samples in the non-muscle invasive recurrent group and 48 urine samples in the no-tumor group.

In the initial analysis recurrence was defined by biopsy verified tumor. But since cystoscopy is not 100% sensitive, we also analyzed the data using combined criteria of either biopsy verified tumor in the bladder or atypical malignant cells grade 2 or 3 in the urine [1,22] as recurrence. Furthermore, follow-up data on the patients was used to make a longitudinal analysis [1,23]. In the statistical analysis we grouped recurrent cases together with the primary cases. In the data analysis we defined positive cytology as presence of atypical cells grade 2 or 3 in the urine (see Additional file 1) [24,25].

### Urine sampling and RNA processing

2 mL of EDTA were added within 15 minutes after urine voiding, and the urine was stored at 4°C for a maximum of two hours until further processing. Then the urine was centrifuged and washed with PBS twice and the cell pellet was lysed in RLT buffer from QIAGEN. Immediately after lysis the samples were stored at minus 80°C. RNA processing was performed using QIAshredder (QIAGEN) and thereafter extracted using the RNeasy mini kit (QIAGEN) and eluted in150 μL RNase free water and stored at minus 80°C. The RNA quality was assessed by use of Eukaryote Total RNA picochips (Agilent Technologies).

### Quantitative polymerase chain reaction

Analysis of *hTERT, SENP1*, and *PPP1CA *was carried out by a combined cDNA and PCR amplification using TaqMan48 from Roche Molecular Systems, Pleasanton, CA. Analysis of the *MCM5 *transcripts was performed in 89 of the 123 samples due to limitation in material. For this we concentrated the remaining RNA using RNeasy MinElute Cleanup kit (QIAGEN) and used Sensiscript RT kit (QIAGEN) for cDNA synthesis using random nonamer primers. QPCR was performed using an ABI 7500 with Taqman probes for MCM5 (Hs00198823_m1). The samples were diluted 1:5 and all samples were analyzed in triplicates (see Additional file 2)

### Statistical analysis

We used Stata 10.0 statistical analysis software (Stata Corporation, College Station, TX, USA) for calculation of ROC curves, nonparametric Wilcoxon rank-sum and Kruskal- Wallis tests for differences in parameters, Log-rank tests for equality of survival function, and Kaplan-Meier survival plots.

## Results

### Characterization of the patients and the urine samples

We collected 123 urine samples from 117 patients during a two year period. The median follow-up time was 28.5 months (range 0-44 months). Including the follow-up period, 77 patients showed tumor recurrence. When comparing the urine samples from primary visits, recurrent visits, and non-recurrent visits we did not find any significant differences in RNA quality, age of the patients, follow up time, nor in previous tumor characteristics (Table 2). Notably, we observed much higher RNA concentrations in primary cancers than in recurrent and non-recurrent cases (p = 0.0001), as well as differences in pellet-size (p = 0.021), in line with earlier studies [1,22,23].

The recurrence risk score for the non muscle-invasive tumors was calculated using EORTC risk tables [26]. We used the previous tumor combined with a history of T1 and T2-4 as a reference to calculate a risk score for recurrence and progression. We found no differences in risk between patients with recurrence and those without recurrence using EORTC risk tables (Table 2).

### Analysis of RT-PCR data using biopsy proven recurrence at sampling

**Figure 1:**
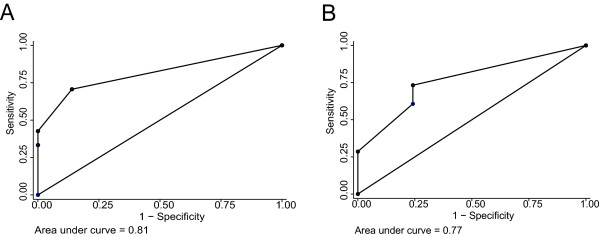
***hTERT *and *MCM5 *ROC curves**. **A**: ROC curve for *hTERT *in combination with cytology grade 2 or grade 3 atypical cells and follow-up information included in the classification. The area under the curve is 81%. **B**: ROC curve for MCM5 in combination with cytology grade 2 or grade 3 atypical cells and follow-up information included in the classification.

We performed quantitative RT-PCR to measure the transcript level of *hTERT, PPP1CA, SENP1*, and *MCM5 *in the urine samples. In the first approach we used the cystoscopy result as the gold standard for recurrence and classified the patients accordingly. After applying optimal cut-off points using ROC curves (examples shown in Figure 1), we observed the following sensitivity and specificity measures for the analyzed markers: *hTERT *(62.5% sensitivity, 72.9% specificity, p < 0.001); *PPP1CA *(68.8% sensitivity and 62.7% specificity, p < 0.001); *SENP1 *(56.3% sensitivity and 78% specificity, p < 0.001); and *MCM5 *(62.5% sensitivity and 65.9% specificity, p = 0.008). Cytology had a 48.3% sensitivity and 79.3% specificity, p = 0.002 (Table 3). We combined the markers with cytology in order to improve our test results and found that especially the combination of *hTERT *and cytology resulted in a sensitivity of 73.3% and a specificity of 74.1% (Table 3).

**Table 3:** Sensitivity and specificity of the urine markers when using cystoscopy as gold standard for detection of bladder cancer

	Sens.	Spec.	AUC	OR	95%CI	PPV	NPV	**P(chi**^**2**^**)**
*hTERT*	62.5	72.9	0.68	4.48	2.09-9.58	71.4	64.2	< 0.001
*PPP1CA*	68.8	62.7	0.66	3.7	1.76-7.77	66.7	64.9	< 0.001
*SENP1*	56.3	78	0.67	4.55	2.08-9.94	73.5	62.2	< 0.001
*MCM5*	62.5	65.9	0.64	3.21	1.36-7.62	68.2	60	0.008
Cytology ALL*	41.7	87.9	0.65	5.2	2.06-13.1	78.1	59.3	< 0.001
Cytology Gr.2-3(Cyt2-3)	48.3	79.3	0.64	3.59	1.6-8	70.7	59.7	0.002
Cyt2-3+hTERT**	73.3	74.1	0.74	7.88	3.49-17.8	74.6	72.9	< 0.001
Cyt2-3+MCM5**	77.8	67.5	0.73	7.27	2.8-18.9	72.9	73	< 0.001
Cyt2-3+ SENP1**	65	74.1	0.70	5.32	2.42-11.7	72.2	67.2	< 0.001
Cyt2-3+PPP1CA**	75	60.3	0.68	4.57	2.09-9.96	66.2	70	< 0.001
Cyt2-3+MCM5+hTERT***	77.8	67.5	0.73	7.27	2.8-18.9	72.9	73	< 0.001

* All atypical cells in the urine recorded as positive.

** One or both positive.

*** If only hTERT was positive then the combined score was classified as negative.

### Analysis of RT-PCR data using follow-up information

In the second approach we included follow-up information and we found that nine of the 16 tumors formerly classified as false positive turned out to be true positive, raising the specificity to 83.7% for *hTERT *(see Table 4). All patients with positive cytology were diagnosed with a tumor in the follow-up period. The median time from urine sampling to tumor diagnosis was 12.3 month for this group (range 3-23). Sensitivity and specificity of all markers using these criteria are listed in Additional file 3. The specificity and especially the sensitivity are higher than observed when only using tumor diagnosis based on cystoscopy information. Furthermore, when we stratified the dataset according to grade, we found a positive correlation between our markers and high grade (Additional file 4).

**Table 4:** Sensitivity and specificity of urine markers when including follow-up data for detection of bladder cancer

	Sensitivity	Specificity	AUC	OR	95% CI	PPV	NPV	P-value
*hTERT*	62	84.1	0.73	8.63	3.47-21.4	87.5	55.2	< 0.001
*MCM5*	61	73.3	0.67	4.3	1.66-11.1	81.8	48.9	0.002
*SENP1*	53.2	84.1	0.69	6	2.43-14.7	85.7	50	< 0.001
*PPP1CA*	64.6	65.9	0.65	3.52	1.63-7.6	77.3	50.9	0.001
Cyt2-3*	42.7	100	0.71			100	50	< 0.001
MCM5+Cyt2-3*	73.2	75.9	0.75	8.59	3.09-23.8	85.4	59.5	< 0.001
hTERT+Cyt 2-3*	70.7	86	0.78	14.9	5.59-39.2	89.8	62.7	< 0.001
hTERT+SENP1**	68.4	75	0.72	6.48	2.85-14.7	83.1	56.8	< 0.001
*hTERT+MCM5*	71.2	66.7	0.69	4.94	1.94-12.6	80.8	54.1	0.001
hTERT+MCM5+Cyt2-3*	73.2	75.9	0.75	8.59	3.09- 23.8	85.4	59.5	< 0.001

* Positive cytology defined by atypical cells grade 2 or 3 in the urine.

**Considered positive if either *SENP1 *or *hTERT *or both are positive.

### Performance compared to cytology and in combination

Interestingly *hTERT*, *MCM5*, and *PPP1CA *were positive in all 7 samples with atypical cells grade 2 or grade 3 that were diagnosed as non-recurrent because no tumors were found at cystoscopy; however, all were diagnosed with a tumor in the follow-up period. We also compared our results to cytology after stratifying the results according to grade using only cystoscopy as the diagnostic criteria. Again we found a positive correlation between grade and sensitivity for all markers and cytology (Additional file 4). The sensitivity was higher for all markers measured in the study compared to cytology, but the highest specificity was observed for cytology. For grade 1 and grade 2 tumors the markers were all superior to cytology. Combination of markers gave the best result for *MCM5 *and *hTERT *with a sensitivity of 76% and a specificity of 77% (Additional file 3). When applying a cut-off with one marker positive it gave a sensitivity of 72% and a specificity of 71%. AUC were 0.72 (Additional file 3).

### Prognostic value at non-recurrent visits

**Figure 2:**
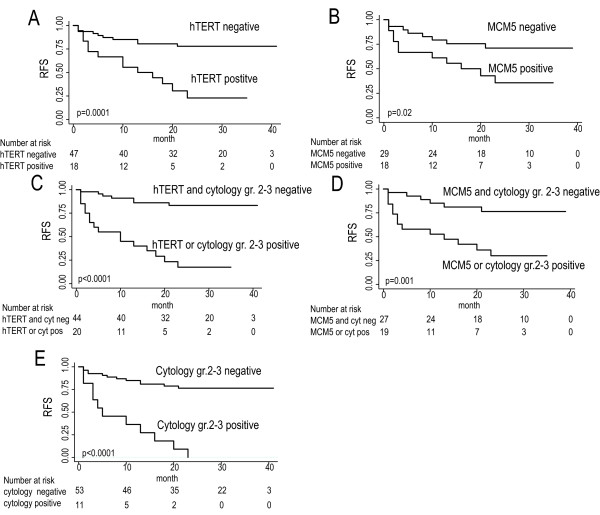
**Recurrence-free survival estimates**. Kaplan-Meier plot of recurrence-free survival for *hTERT *(N = 65) (**A**) and *MCM5 *(N = 47) (**B**) alone and in combination with cytology (**C **and **D**). **E**: Kaplan-Meier plot of recurrence-free survival based on cytology results. Only urine samples from patients with no cystoscopy detectable tumors are included.

We evaluated the prognostic value of the markers at non-recurrent visits. We found that especially *hTERT *and *MCM5 *expression were able to significantly add information to the diagnosis obtained by cystoscopy and cytology as seen in Figure 2. The most significant difference was observed for *hTERT*, where 75% of patients who were *hTERT *positive without a tumor at cystoscopy recurred within 24 months. In contrast only 25% of those who were *hTERT *negative and had no tumor at cystoscopy recurred within 24 months. The same tendency was found for *MCM5*. Fifty-six percent (9/16) of the positives (at first analysis considered false positive) recurred shortly after urine sampling and when we considered the disease course in the data analysis, they ended up as true positive. Univariate Cox regression analysis also showed that both *hTERT *and *MCM5 *were significantly associated with recurrence free survival (*hTERT*: HR = 4.6, p < 0.001; *MCM5*: HR = 2.7, p = 0.03). None of the risk factors for recurrence (i.e. previous stage, grade, size, multiplicity and CIS) were significantly associated with recurrence free survival in this group of patients. Multivariate Cox regression analysis showed that *hTERT *and *MCM5 *were independent prognostic markers for recurrence free survival when stratifying for previous grade, multiplicity and CIS (*hTERT*: HR = 8.5, p < 0.001; *MCM5*: HR = 4.2, p = 0.02). No other variables were significantly associated with recurrence free survival in the multivariate analyses. The lack of association between the risk factor for recurrence and the later tumor recurrence events is probably observed because of the limited number of urine samples included in this analysis (non-recurrent visits only).

## Discussion

Finding a way to diagnose bladder cancer recurrence using a urine marker has been a major challenge in many years. Here we aimed at making a simple test useful on urine samples from every patient. A cross section of patients was used from the clinic disregarding large differences in disease courses and earlier treatment.

The most well characterized marker in this study was *hTERT*. The urine expression has been studied previously and impressive results with sensitivity levels of 92% and specificity of 96% have been reported. In contrast to this study, the patient population consisted only of untreated bladder cancer patients in which a higher RNA concentration was expected in the urine [6] and thereby a higher detection rate [23]. A urinary marker also needs to be insensitive to benign conditions like infections and cystolithiasis. Melissourgos *et al. *used a control group of 128 non-bladder cancer patients; false positive cases of *hTERT *detection occurred in persons with benign prostate hyperplasia or cystolitiasis [6]. Cytology has an equivalent false positive rate, but the false positives were associated with bladder inflammation [6]. Surprisingly, *hTERT *mRNA is not detected in cases where lymphocyte inflammation is present, since telomerase enzymatic activity is up regulated in activated lymphocytes. However, this enzymatic up-regulation may just be a transient effect [6,8]. Another large study of *hTERT *and *hTR *in urine of 465 subjects in three different groups, bladder cancer, benign urologic conditions and healthy controls has been conducted by Weikert *et al. *[8]. Previous studies reported sensitivity between 55.2- 91.8% and specificity between 85-96.1%. We observed a comparable sensitivity of 62%, but the specificity of 84.1% is a little lower [6,7]. This could be due to the high number of low-grade Ta tumors in this study. The data presented here gives hope for a non-invasive test able to improve the current diagnostic regime, but there are some problems when working with urine RNA, as it is highly degradable and variation in cell content is large. We compared the RNA concentration in the different samples and found a significant difference, when comparing the primary group, the recurrent group and the non-recurrent group. The primary group had a much higher amount of RNA as compared to the other groups. The non-recurrence and recurrence groups are rather similar in RNA concentration. Van Rhijn *et al. *reported lower biomarker sensitivity in patients who are under surveillance for bladder cancer compared to a cross sectional sampling of primary and surveillance patients, while their specificity was not influenced [23]. In accordance with our results the sensitivity increases with higher tumor grade. Okumura *et al. *2004 found the opposite correlation between *hTERT *and tumor grade and suggested an alternative mechanism that can immortalize cells [21]. *SENP1 *in our study was included based on the same theory, since it is known that in certain cancers maintain their telomeres by a telomerase independent mechanism known as alternative lengthening of telomeres (ALT) [27,28]. We examined the possibility to increase the test using a molecular marker for ALT in conjunction with expression of *hTERT*. *SENP1 *might serve as such a marker. We found that *SENP1 *was positive in *hTERT *negative samples. Unfortunately diagnostic power of the combined analysis did not increase compared to *hTERT *alone (Additional file 4).

## Conclusions

With *hTERT *we should in theory have a marker unique to tumor cells; while gaining telomerase function they become immortal and insensitive to the normal senescence and apoptosis signal. Normally, *hTERT *is not seen in normal cells apart from germ- and stem cells. *SENP1 *was included as a potential marker of alternative lengthening of telomeres in the *hTERT *negative, but immortalized cells and it was a significant biomarker in our study. *MCM5 *has been characterized as highly expressed in high grade tumors. Telomerase has been suggested to be present in low grade tumors and these two markers should, in theory, complement each other. However, we did not observe an increase in accuracy of the markers when combined.

In conclusion, the investigated candidates are promising but all have a lower specificity than cytology. They have a better sensitivity, and a combination with cytology may be an alternative to existing methods.

## Competing interests

Cherie Holcomb is an employee Roche Molecular Systems, Pleasanton California; Roche Molecular Systems is a funding supporter.

The authors declare that they have no other competing interests.

## Authors' contributions

ASBE and KZ reviewed the patients' history and pathological data, performed sample selection and retrieval, carried out the molecular analyses, performed data analysis and part of the statistical analyses, interpretation of data. ASBE drafted the manuscript. HT collected the urine samples, and optimized the methods used. PPM, CH and FM participated in the method selection and interpretation of data, and MB was responsible for the clinical database. TFØ, CH, RH, HT and LD participated in the conception and design of the study, and critically revised the manuscript. LD supervised the analysis and interpretation of the data, and helped to draft the manuscript. All authors read and approved the final manuscript.

## Pre-publication history

The pre-publication history for this paper can be accessed here:

http://www.biomedcentral.com/1471-2407/10/646/prepub

## Supplementary Material

Additional file 1 **Clinical data for the patients in the study**. Click here for file

Additional file 2 **Data for the examined urines in the study**. Click here for file

Additional file 3 **Sensitivity and specificity when using a combined tumor diagnosis using both cystoscopy verified recurrence and atypical cells grade 2 or 3 in the urine**. Click here for file

Additional file 4 **Marker sensitivity and specificity when stratified for tumor grade**. Click here for file

## References

[B1] Van Tilborg AA Bangma CH Zwarthoff EC Bladder cancer biomarkers and their role in surveillance and screening Int J Urol 2009 16 1 23 30 10.1111/j.1442-2042.2008.02174.x 19120523

[B2] Lotan Y Roehrborn CG Sensitivity and specificity of commonly available bladder tumor markers versus cytology: results of a comprehensive literature review and meta-analyses Urology 2003 61 1 109 118 discussion 118 10.1016/S0090-4295(02)02136-2 12559279

[B3] Hong YM Loughlin KR Economic impact of tumor markers in bladder cancer surveillance Urology 2008 71 1 131 135 10.1016/j.urology.2007.08.014 18242381

[B4] Bennett A Telomerase and other novel approaches to bladder cancer detection Clin Lab Sci 2008 21 3 185 190 quiz 191-182, following 192 18678141

[B5] Bialkowska-Hobrzanska H Bowles L Bukala B Joseph MG Fletcher R Razvi H Comparison of human telomerase reverse transcriptase messenger RNA and telomerase activity as urine markers for diagnosis of bladder carcinoma Mol Diagn 2000 5 4 267 277 11172490 10.1007/BF03262087

[B6] Melissourgos N Kastrinakis NG Davilas I Foukas P Farmakis A Lykourinas M Detection of human telomerase reverse transcriptase mRNA in urine of patients with bladder cancer: evaluation of an emerging tumor marker Urology 2003 62 2 362 367 10.1016/S0090-4295(03)00254-1 12893365

[B7] Takihana Y Tsuchida T Fukasawa M Araki I Tanabe N Takeda M Real-time quantitative analysis for human telomerase reverse transcriptase mRNA and human telomerase RNA component mRNA expressions as markers for clinicopathologic parameters in urinary bladder cancer Int J Urol 2006 13 4 401 408 10.1111/j.1442-2042.2006.01300.x 16734859

[B8] Weikert S Krause H Wolff I Christoph F Schrader M Emrich T Miller K Muller M Quantitative evaluation of telomerase subunits in urine as biomarkers for noninvasive detection of bladder cancer Int J Cancer 2005 117 2 274 280 10.1002/ijc.21168 15900578

[B9] Shay JW Telomerase in human development and cancer J Cell Physiol 1997 173 2 266 270 10.1002/(SICI)1097-4652(199711)173:2<266::AID-JCP33>3.0.CO;2-B 9365534

[B10] Harley CB Kim NW Prowse KR Weinrich SL Hirsch KS West MD Bacchetti S Hirte HW Counter CM Greider CW Telomerase, cell immortality, and cancer Cold Spring Harb Symp Quant Biol 1994 59 307 315 7587082 10.1101/sqb.1994.059.01.035

[B11] Kim NW Piatyszek MA Prowse KR Harley CB West MD Ho PL Coviello GM Wright WE Weinrich SL Shay JW Specific association of human telomerase activity with immortal cells and cancer Science 1994 266 5193 2011 2015 10.1126/science.7605428 7605428

[B12] Morin GB The human telomere terminal transferase enzyme is a ribonucleoprotein that synthesizes TTAGGG repeats Cell 1989 59 3 521 529 10.1016/0092-8674(89)90035-4 2805070

[B13] Stoeber K Halsall I Freeman A Swinn R Doble A Morris L Coleman N Bullock N Laskey RA Hales CN Immunoassay for urothelial cancers that detects DNA replication protein Mcm5 in urine Lancet 1999 354 9189 1524 1525 10.1016/S0140-6736(99)04265-8 10551502

[B14] Korkolopoulou P Givalos N Saetta A Goudopoulou A Gakiopoulou H Thymara I Thomas-Tsagli E Patsouris E Minichromosome maintenance proteins 2 and 5 expression in muscle-invasive urothelial cancer: a multivariate survival study including proliferation markers and cell cycle regulators Hum Pathol 2005 36 8 899 907 10.1016/j.humpath.2005.06.008 16112007

[B15] Castro ME Ferrer I Cascon A Guijarro MV Lleonart M Ramon y Cajal S Leal JF Robledo M Carnero A PPP1CA contributes to the senescence program induced by oncogenic Ras Carcinogenesis 2008 29 3 491 499 10.1093/carcin/bgm246 18204081

[B16] Prowatke I Devens F Benner A Grone EF Mertens D Grone HJ Lichter P Joos S Expression analysis of imbalanced genes in prostate carcinoma using tissue microarrays Br J Cancer 2007 96 1 82 88 10.1038/sj.bjc.6603490 17146477 PMC2360197

[B17] Ohbayashi N Kawakami S Muromoto R Togi S Ikeda O Kamitani S Sekine Y Honjoh T Matsuda T The IL-6 family of cytokines modulates STAT3 activation by desumoylation of PML through SENP1 induction Biochem Biophys Res Commun 2008 371 4 823 828 10.1016/j.bbrc.2008.04.179 18474224

[B18] Everett RD Lomonte P Sternsdorf T van Driel R Orr A Cell cycle regulation of PML modification and ND10 composition J Cell Sci 1999 112 Pt 24 4581 4588 10574707 10.1242/jcs.112.24.4581

[B19] Bernardi R Pandolfi PP Structure, dynamics and functions of promyelocytic leukaemia nuclear bodies Nat Rev Mol Cell Biol 2007 8 12 1006 1016 10.1038/nrm2277 17928811

[B20] Yeager TR Neumann AA Englezou A Huschtscha LI Noble JR Reddel RR Telomerase-negative immortalized human cells contain a novel type of promyelocytic leukemia (PML) body Cancer Res 1999 59 17 4175 4179 10485449

[B21] Okumura A Mizuno I Nagakawa O Fuse H Telomerase activity is correlated with lower grade and lower stage bladder carcinomas Int J Urol 2004 11 12 1082 1086 10.1111/j.1442-2042.2004.00960.x 15663679

[B22] Kinoshita H Ogawa O Kakehi Y Mishina M Mitsumori K Itoh N Yamada H Terachi T Yoshida O Detection of telomerase activity in exfoliated cells in urine from patients with bladder cancer J Natl Cancer Inst 1997 89 10 724 730 10.1093/jnci/89.10.724 9168188

[B23] van Rhijn BW van der Poel HG van der Kwast TH Urine markers for bladder cancer surveillance: a systematic review Eur Urol 2005 47 6 736 748 10.1016/j.eururo.2005.03.014 15925067

[B24] Harving N Petersen SE Melsen F Wolf H Urinary cytology in the detection of bladder tumours. Influence of concomitant urothelial atypia Scand J Urol Nephrol Suppl 1989 125 127 131 2633311

[B25] Harving N Wolf H Melsen F Positive urinary cytology after tumor resection: an indicator for concomitant carcinoma in situ J Urol 1988 140 3 495 497 3411659 10.1016/s0022-5347(17)41700-9

[B26] Sylvester RJ van der Meijden AP Oosterlinck W Witjes JA Bouffioux C Denis L Newling DW Kurth K Predicting recurrence and progression in individual patients with stage Ta T1 bladder cancer using EORTC risk tables: a combined analysis of 2596 patients from seven EORTC trials Eur Urol 2006 49 3 466 465 discussion 475-467 10.1016/j.eururo.2005.12.031 16442208

[B27] Bryan TM Englezou A Gupta J Bacchetti S Reddel RR Telomere elongation in immortal human cells without detectable telomerase activity EMBO J 1995 14 17 4240 4248 7556065 10.1002/j.1460-2075.1995.tb00098.x PMC394507

[B28] Johnson JE Broccoli D Telomere maintenance in sarcomas Curr Opin Oncol 2007 19 4 377 382 10.1097/CCO.0b013e3281214423 17545803

