# Supplementary material for: Prediction and diagnosis of bladder cancer recurrence based on urinary content of hTERT, SENP1, PPP1CA, and MCM5 transcripts
Source: BMC Cancer. 2010 Nov 24;10:646. doi: 10.1186/1471-2407-10-646 (PMC3001447; doi:10.1186/1471-2407-10-646)
Supplement: Additional file 3 — Sensitivity and specificity when using a combined tumor diagnosis using both cystoscopy verified recurrence and atypical cells grade 2 or 3 in the urine. [file 1471-2407-10-646-S3.DOC]

# Additional file 3

Summary of the sensitivity and specificity results obtained when including cytology results in the diagnosis of tumors at sampling. *hTERT, SENP1*, *PPP1CA* was measured in 123 urine samples, whereas *MCM5* was measured in 89 samples.

|  | **Positive/**  **Total** | **Sens** | **Spec** | **AUC** | **OR** | **95 ci** | **PPV** | **NPV** | **P(chi2)** |
| --- | --- | --- | --- | --- | --- | --- | --- | --- | --- |
| ***hTERT*** | 56/123 | 66.2 | 82.7 | 0.74 | 9.36 | 3.96-22.1 | 83.9 | 64.2 | <0.001 |
| ***MCM5*** | 44/89 | 66.7 | 77.1 | 0.72 | 6.75 | 2.59-17.5 | 81.8 | 60 | <0.001 |
| ***PPP1CA*** | 70/123 | 73.2 | 65.4 | 0.69 | 5.17 | 2.39-11.2 | 74.3 | 64.2 | <0.001 |
| ***SENP1*** | 58/123 | 63.4 | 75 | 0.69 | 5.19 | 2.37-11.4 | 77.6 | 60 | <0.001 |
| ***MCM5+hTERT*** | 49/89 | 75.9 | 77.1 | 0.77 | 10.6 | 3.94-28.7 | 83.7 | 67.5 | <0.001 |
| ***MCM5+hTERT+PPP1CA*** | 48/89 | 74.1 | 77.1 | 0.76 | 9.64 | 3.6-25.8 | 83.3 | 65.9 | <0.001 |
| ***hTERT+PPP1CA*** | 60/123 | 70.4 | 80.8 | 0.76 | 10 | 4.28-23.3 | 83.3 | 66.7 | <0.001 |
| ***MCM5+hTERT+SENP1*** | 49/89 | 75.9 | 77.1 | 0.77 | 10.6 | 3.94-28.7 | 83.7 | 67.5 | <0.001 |
| ***MCM5+PPP1CA*** | 56/89 | 77.8 | 60 | 0.69 | 5.25 | 2.09-13.2 | 75 | 63.6 | <0.001 |
| ***SENP1+hTERT*** | 51/123 | 71.8 | 71.2 | 0.72 | 6.29 | 2.86-13.8 | 77.3 | 64.9 | <0.001 |
