# Supplementary material for: Prediction and diagnosis of bladder cancer recurrence based on urinary content of hTERT, SENP1, PPP1CA, and MCM5 transcripts
Source: BMC Cancer. 2010 Nov 24;10:646. doi: 10.1186/1471-2407-10-646 (PMC3001447; doi:10.1186/1471-2407-10-646)
Supplement: Additional file 4 — Marker sensitivity and specificity when stratified for tumor grade. [file 1471-2407-10-646-S4.DOC]

# Additional file 4:

Summary of the sensitivity and specificity results obtained when stratified for tumor grade. Cystoscopy alone is used in the upper part of the table and cystoscopy and cytology in combination is used in the lower part of the table as gold standard for detection of bladder cancer.

*grade 2-3 atypical cells

| **Stratified for grade** | **hTERT** | **MCM5** | **SENP1** | **PPP1CA** | **Cytology** |
| --- | --- | --- | --- | --- | --- |
| **Using only cystoscopy as determination of bladder cancer** | | | | | |
| **Grade 1** | 0.4 | 0.2 | 0.4 | 0.5 | 0.2 |
| **Grade 2** | 0.57 | 0.64 | 0.46 | 0.64 | 0.22 |
| **Grade 3** | 0.77 | 0.64 | 0.54 | 0.65 | 0.74 |
| **Overall sensitivity** | 0.63 | 0.63 | 0.48 | 0.63 | 0.42 |
| **Overall specificity** | 0.73 | 0.59 | 0.69 | 0.59 | 0.88 |
| **Using both cytology and cystoscopy as determination of bladder cancer** | | | | | |
| **positive cytology* only** | 1 | 0.71 | 1 | 1 | ----- |
| **Grade 1** | 0.4 | 0.4 | 0.43 | 0.6 | 0.3 |
| **Grade 2** | 0.57 | 0.54 | 0.6 | 0.61 | 0.3 |
| **Grade 3** | 0.71 | 0.81 | 0.75 | 0.85 | 0.87 |
| **Overall sensitivity** | 0.66 | 0.63 | 0.67 | 0.73 | 0.57 |
| **Overall specificity** | 0.83 | 0.75 | 0.52 | 0.65 | 0.98 |
